# Supplementary material for: Social Participation and Loneliness in Older Adults in a Rural Australian Context: Individual and Organizational Perspectives
Source: Int J Environ Res Public Health. 2024 Jul 8;21(7):886. doi: 10.3390/ijerph21070886 (PMC11276585; doi:10.3390/ijerph21070886)
Supplement: Supplementary file 1 [file ijerph-21-00886-s001.zip › ijerph-3080267-supplementary.pdf]

Supplementary File S1:

Part One: In-Depth Interview Guide

This document is only for investigators.

This interview is for participants who score with high loneliness (4 or above on the DeJong Gierveld Loneliness Scale). Researchers will begin with reflecting feelings back to participants, and then ask participants a few questions about their emotions, coping, and social participation.

Researcher: *I see that you might be feeling like..*

1) You miss having people around
2) You're lacking someone close to you
3) You might be feeling disconnected

Would it be alright if we asked you some followup questions now or at a later time?

Can you please introduce yourself, and tell me about which groups or programs you attend in Carnarvon?

Previous studies have shown that there are a few ways to help with loneliness. We're going to ask you some questions about common programs and services used to target feelings of loneliness. Some of these include groups, one-on-one support, and therapy.

Groups

1. Can you tell me your opinions on participating in groups?
2. Can you tell me about the groups you're a part of in Carnarvon?
    a. How did you hear about it?
    b. How do you travel to get there?
    c. What makes you keep going?
    d. What is your favorite part?
    e. Least favorite part?
    f. Anything you would change?
3. Have there been any groups you stopped going to?
    a. Why?
4. **If they have not been to any purely social clubs**:
    a. Have you participated in any social clubs, where the main focus was just to make friends (e.g. bingo, morning tea)?
    b. Can you tell me why you stopped?
    c. Can you tell me why you're not interested?/why you haven't gone?
5. **If they have not been to any common interest groups**:
    a. Have you participated in any common interest groups, where people learn a new

skill together or share a hobby?

    b. Can you tell me about why you stopped?
    c. Can you tell me about why you're not interested?/ why you haven't gone?

6. Can you tell me about a group you *don't* attend?
    a. What makes you not (want to) attend this group?
    b. What would need to change for you to be able to attend?
7. What kinds of groups would you be interested in attending?
    a. Common interest vs. social, Big vs. small, New skill vs. hobby, Intellectual vs. active, etc.
8. What might make a program or activity appealing to you or other people in your age group?
9. What do you think makes a program less appealing or successful?

Befriending

10. Can you tell me your opinions on participating in one-on-one support (e.g. companion programs)?
    a. Would you participate if these were available?

Therapy

11. Do you know of any support animal organizations or therapy services?
    a. If yes: do you use any of them? What are some reasons…?
    b. If no: would you participate if these were available? What are some reasons…?

Misc

12. Do you feel connected to your community?
    a. What makes you feel that way?
13. What do you think might be able to help with feeling alone or disconnected? What has helped you?
14. Have issues with mobility ever made it difficult for you to participate in a program?
15. Have issues with technology ever made it difficult for you to participate in a program?

a. Have you ever thought about the library…

Talking about it

16. Have you talked about these feelings with..
    a. GP?
    b. Therapist?
    c. Friend?
    d. Family?

17. Do you ever worry about telling people if you're lonely?
    a. If so, why?

Opportunity

I understand you feel that services and groups aren't available/suitable to your needs. We've been hearing this a lot. We would like your opinions on some ideas to make it better.

18. How do you think that organizations in Carnarvon, or the Shire could help with this problem?
    a. Have you seen any efforts to help with loneliness?

\

Supplementary File S2:

Interview Guide for Part Two

Project title: Social Connection and Participation in Older People in a Regional Setting
Name of Researchers: Dr. Sandra Thompson, Leah Wilson, Carrigan Rice, Jillian Bilcich, Kathryn Fitzgerald, and Josephine Parry

Background:

The purpose of this research is to explore social relationships and barriers to social participation in older populations. Previous research has shown that social networks and connectedness tend to decrease with aging and are associated with feelings of loneliness and isolation. It is known that these feelings are associated with poor mental and physical health. Interventions targeting loneliness and social isolation have had mixed results. Research suggests that a holistic community approach is more effective in targeting feelings of loneliness and social isolation. Unfortunately there has been little research into this topic in rural and more remote areas, and we have not identified any from rural and remote Western Australia. Our work aims to conduct an environmental scan on the approaches and resources available in Carnarvon, and to identify barriers for social participation reported by older Carnarvon residents. Through gathering the perspectives of local older residents and those of service providers, we will be mapping and gapping current resources, utilizing a collaborative approach. The benefit of this research will be that your program and other organizations in Carnarvon can use the results to better meet the needs of older adults.

Instructions:

Please answer these questions to the best of your ability. This survey is designed for directors or staff who have general knowledge of their organization. The questions will take approximately 30 minutes to complete. Participation in this survey is voluntary and no personally identifiable data will be used in response analysis or shared with anyone outside the research team without express consent. Please note that there are multiple types of organizations assessed in this questionnaire, however you will only answer questions pertaining to your type of organization.

Responses from the questionnaire will be explored for themes using the Promising Approaches Framework, a tool to promote healthy aging and aid in the prevention of loneliness and social isolation. If you have any questions, please follow up with Leah Wilson, research assistant from Georgetown University, by email 00113255@uwa.edu.au.

Interview Guide

Name:
Title/occupation:
Organization/agency name:
Email:

Phone:

1. Can you tell us a little about your organization and the services your organization provides?
   Follow-up questions:
   a.) Does your service target a specific demographic?
   b.) What age group uses your services the most?
   c.) How is your organization funded? Is that funding for a specific target? d.) What might motivate someone to come to your organization or use your service? e.) How do you attract new people to your organization?
   f.) Which services, if any, are aimed at older people?

2. How is your organization run?
   Follow-up questions:
   a. How often do you meet? What days and times are your services available? b. Does your organization have any managing member(s) over the age of 50? c. How has your organization changed over the past 10 years?
   d. Are you interested in, or do you have plans to, expand?

3. If any, which other programs do you work closely with?

4. Can you tell us about how people join your group or use your service?
   Follow up questions:
   a. How much does it cost to use your service?
   b. How might people contact you to access your service?
   c. How might people travel to use your service?
   d. Does your organization provide digital ways to utilize your service?
   e. Could someone with limited mobility attend your group? (e.g. wheelchair access, handrails, etc.)

5. Based on your experience, do you believe that your organization encounters lonely or socially isolated people?
   Follow up questions:
   a. How might you identify someone who is lonely or socially isolated?
   b. Has anyone confided in you that they are lonely?
      i. What kinds of people confide in you for that?
   c. What have you heard anything about loneliness in Carnarvon?
   d. What sorts of programs might you connect lonely or socially isolated people to?
6. In your experience, what might stop someone from joining your organization/using your service?
   a. Is there anyone responsible for reaching out to members, for example if someone

doesn’t show up for a while?

7. Do you feel like you or other staff would be interested in going to a loneliness training?
    a. What factors would stop you from coming?

8. We are using the Promising Approaches Framework and it defines a few different types of organizations and social groups. This includes connector services, group participation programs, psychological services, and services that target barriers to participation. *Here is a sheet that describes these services.* Which of the following best describes your organization?
    - ☐ Connector service or resource that reaches out and connects people with further resources (e.g. health, employment, social service, government, etc.)
    - ☐ Service that reduces barriers to connection (e.g. digital access hub, public transportation, mobility assistance)
    - ☐ Service that provides therapy, counseling, or other psychological intervention
    - ☐ Activity or social group
    - ☐ System approaches are organizations that can develop interventions or groups to address identified needs in a specific group (e.g. local government, faith and volunteering organizations, hospitals)

We have a few more questions targeting these specific categories.

Connector service

9. What is the most common service you provide to people over 50?

10. When connecting people to services and/or for additional help, how do you determine if your resources are appropriate for the person? How do you consider..
    a. ☐Transport
    b. ☐Digital access
    c. ☐Mobility
    d. ☐Financial resources
    e. ☐Cultural appropriateness
    f. ☐Safety
    g. ☐Individual needs (as compared to uniform services)

11. If someone has a challenge using or accessing your resource, how do you help them?
    a. Can you provide an example?
12. When connecting someone to a service, do you provide emotional support? Do you feel comfortable/qualified to do so?

13. Do you think that the communities you see feel comfortable asking you for emotional support?

14. Within your organization, do you provide follow up after connecting people to resources?

15. Based on your experience and work in Carnarvon, what service gaps might exist in Carnarvon for individuals experiencing loneliness and social isolation?

16. What are Carnarvon's strengths?

Barrier-reducing services

1. What is the most common service you provide to people over 50?

2. How does your service reduce barriers to connections?

3. Based on your experience and work in Carnarvon, what service gaps might exist in Carnarvon for individuals experiencing loneliness and social isolation?

4. What are Carnarvon's strengths?

Psych services:

1. What type of psychological intervention(s) do you provide?

2. What is the most common service you provide to people over 50?

3. If you identified someone as lonely or socially isolated, what type of intervention might you provide?

4. Does your service provide education about loneliness and social isolation?

5. Based on your experience and work in Carnarvon, what service gaps might exist in Carnarvon for individuals experiencing loneliness and social isolation?

6. What are Carnarvon's strengths?

Social/Activity Groups:

1. Please indicate which of these describe your group.

- ☐ Sport or physical activity
- ☐ Arts or creative activity
- ☐ Social group
- ☐ Sharing identity or experience(explain)
- ☐ Food
- ☐ Helping out or making change(advocacy group or volunteer)
- ☐ Learning new skill
- ☐ Spiritual

2. Please indicate which of these describes how members get involved in your group.
    - ☐ Informal socializing
    - ☐ Volunteering
    - ☐ Hold formal role
    - ☐ Participating in something or formal meeting
    - ☐ Learning together(example: class learning)
    - ☐ Peer-to-peer/reciprocal support (example: support group)

3. How do you attract new people to your group?

4. What might motivate people to stay in your group?

5. Is there anyone responsible for reaching out to members, for example if someone doesn't show up for a while?

6. Based on your experience and work in Carnarvon, what service gaps might exist in Carnarvon for individuals experiencing loneliness and social isolation?

7. What are Carnarvon's strengths?

System approaches

1. Does your organization have any leadership members developing programs that are over the age of 50?
    a. Over 70?

2. Do you have any programs that target people over the age of 50? a.
    Or individuals who are lonely?

3. How do/did you determine the need for a program?
    a. How are these programs informed?

4. When creating programs, were populations in need included in development?

5. When developing programs, how do you look at resources in the community already available?

6. When creating programs, how is collaboration involved?

7. When creating programs, how do you determine if your program/intervention is appropriate for the group in need? How do you consider..
    a. ☐ Transport
    b. ☐ Digital access
    c. ☐ Mobility
    d. ☐ Financial resources
    e. ☐ Cultural appropriateness
    f. ☐ Safety
    g. ☐ Individual needs (as compared to uniform services)

8. Based on your experience and work in Carnarvon, what service gaps might exist in Carnarvon for individuals experiencing loneliness and social isolation?

9. What are Carnarvon's strengths?
